# Supplementary material for: The Growth of Graphene on Ni–Cu Alloy Thin Films at a Low Temperature and Its Carbon Diffusion Mechanism
Source: Nanomaterials (Basel). 2019 Nov 17;9(11):1633. doi: 10.3390/nano9111633 (PMC6915519; doi:10.3390/nano9111633)
Supplement: Supplementary file 1 [file nanomaterials-09-01633-s001.pdf]

Supplementary Materials: The Growth of Graphene on Ni–Cu Alloy Thin Films at a Low Temperature and Its Carbon Diffusion Mechanism

Yibo Dong [1], Sheng Guo [2], Huahai Mao [3,4], Chen Xu [1,*], Yiyang Xie [1], Chuantong Cheng [5], Xurui Mao [5], Jun Deng [1], Guanzhong Pan [1] and Jie Sun [1,6,*]

1. Key Laboratory of Optoelectronics Technology, College of Microelectronics, Beijing University of Technology, Beijing 100124, China; donyibo@emails.bjut.edu.cn (Y.D.); xieyiyang@bjut.edu.cn (Y.X.); dengsu@bjut.edu.cn (J.D.); guanzhongpan@emails.bjut.edu.cn (G.P.)
2. Department of Industrial and Materials Science, Chalmers University of Technology, 41296 Gothenburg, Sweden; sheng.guo@chalmers.se
3. Materials Science and Engineering, KTH Royal Institute of Technology, Brinellvägen 23, 10044 Stockholm, Sweden; huahai@kth.se
4. Thermo-Calc Software AB, Råsundavägen 18, 16967 Solna, Sweden
5. State Key Laboratory of Integrated Optoelectronics, Institute of Semiconductor, Chinese Academy of Sciences, Beijing 100083, China; chengchuantong@semi.ac.cn (C.C.); maoxurui@semi.ac.cn (X.M.)
6. Quantum Device Physics Laboratory, Department of Microtechnology and Nanoscience, Chalmers University of Technology, 41296 Gothenburg, Sweden

* Correspondence: xuchen58@bjut.edu.cn (C.X.); jie.sun@chalmers.se (J.S.)

1. The growth equipment used is a vertical cold wall CVD furnace (Black Magic, Aixtron). Figure S1a shows the schematic illustration of the equipment. Figure S1b and c are the shematic illustrations of the graphene growth process and its different stages.

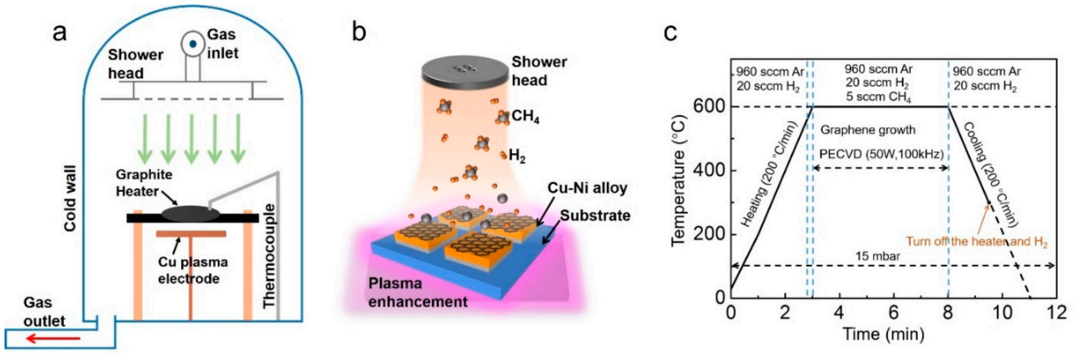

Figure S1. (**a**) Schematic illustration of the graphene growth equipment (vertical cold wall CVD furnace). (**b**) Schematic illustration of the graphene growth. During the growth process, the substrate is placed on the graphite heater, and the Cu plasma electrode is used to provide plasma enhancement. (**c**) Schematic illustration of the graphene growth process and its different stages.

2. Figure S2 a and b are the SEM images of 300 nm Ni and Cu film respectively after graphene growth at 800 °C. We can see that the average grain size of Ni is obviously smaller than that of Cu, indicating that the grain boundaries of Ni film is more than that of Cu. Graphene generally nucleates at grain boundaries, so it can be inferred that the density of graphene nucleation point on Ni surface should be higher than that of Cu.

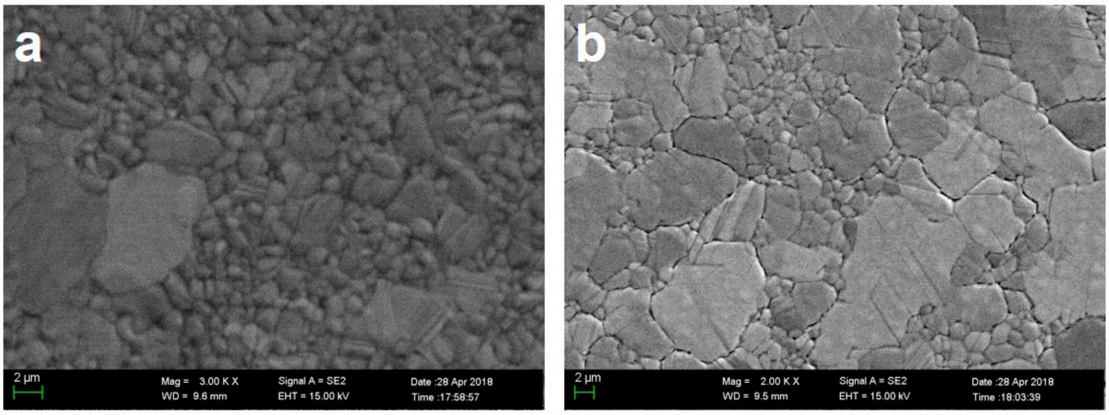

Figure S2. SEM images of Ni (**a**) and Cu (**b**) films respectively after the growth of graphene.

3. As shown in Figure S3, we used pure Ni as catalyst to grow graphene without plasma. The carbon source used is methane. The growth time is 45 s. We studied different growth temperatures (from 550 °C to 375 °C). Graphene can be observed directly on 300 nm SiO_2/Si substrate by optical microscopy [1]. It can be seen from the figures, when the temperature drops to 450 °C, graphene can not be observed on the surface of the samples, so we can conclude that the lowest growth temperature of graphene catalyzed by Ni without plasma is around 450 °C.

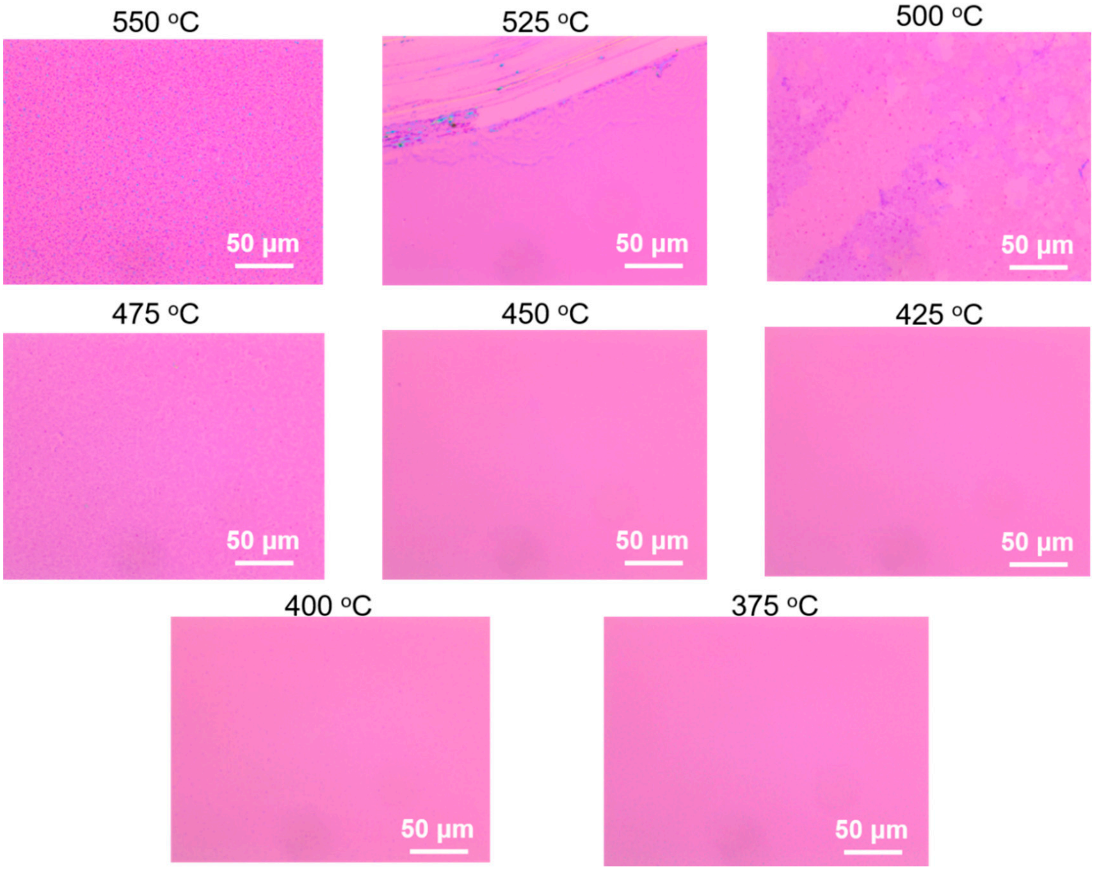

Figure S3. Optical images of the Ni-catalyzed graphene grown at different temperatures. When the growth temperature is lower than or equal to 450 °C, the surface of the obtained sample has no graphene growth, which indicates that the lowest growth temperature of Ni-catalyzed graphene is about 450 °C.

4. We used a very simple and intuitive method to prove the etching effect of hydrogen plasma on graphite. As shown in Figure S4, we used a plasma electrode made of Cu, which shows the color of Cu before growth. The ratio of hydrogen/methane we used is 20 sccm/20 sccm. After several times of growth, the Cu plasma electrode was covered with a very thick layer of graphite, resulting in black color. After that, we used hydrogen plasma to etch the Cu electrode. The etching conditions are as follows: hydrogen flow rate 1000 sccm, temperature 600 °C, plasma power 400 W with a time of 20 min. After etching, it can be seen that the graphite on the surface of Cu plasma electrode has been completely etched, and the original color of Cu has been restored. This experiment is an intuitive proof that the effect of the two gases (the growth effect of methane and the etching effect of hydrogen) are greatly accelerated by the plasma enhancement.

At the same time, through the color of Cu plasma electrodes, we can also see a very intuitive phenomenon, that is, when hydrogen: methane=20 sccm:20 sccm, after many times of growth, the surface of Cu plasma electrodes will have a very thick graphite coating and show black color. However, when hydrogen: methane=20 sccm:5 sccm, the surface of Cu plasma electrode can remain relatively clean after many times of growth. This shows that the growth of graphite induced by methane plasma and the etching of graphite induced by hydrogen plasma are dynamic processes. When hydrogen: methane=20 sccm:20 sccm, the growth rate of graphite is faster than that of etching. When hydrogen: methane=20 sccm:5 sccm, the growth rate of graphite is slower than that of etching. As for the quality of the graphene grown under the two different flow rates, it was found that there was no significant difference.

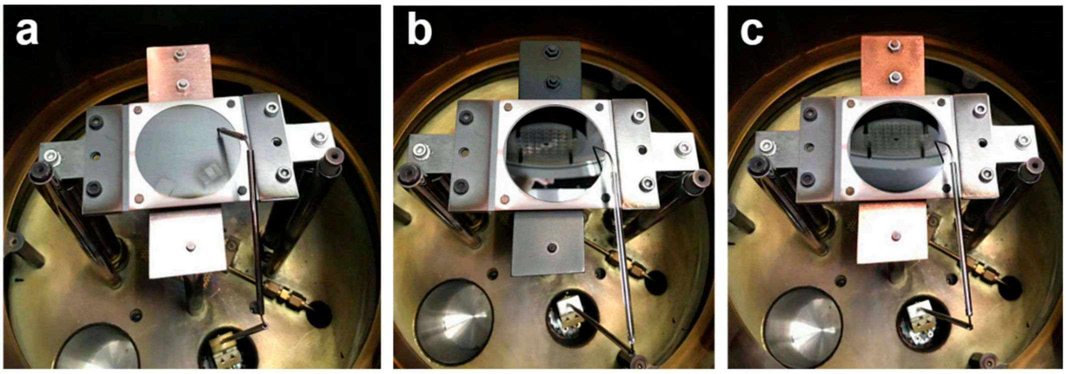

Figure S4. Digital images of the growth chamber before graphene growth (**a**), after several times of graphene growth (**b**) and after H2 plasma etching (**c**).

5. Selection of Alloys for Low Temperature Growth

(1) High catalytic activity

According to Ref. 32 in the paper, the catalytic activity order of these metals is found to be Ni ≈ Rh ≈ Co ≈ Ru > Pd ≈ Pt ≈ Ir > Cu > Au

Conclusion: Au should be excluded due to its low catalytic activity.

(2) Fast carbon diffusion in the metal

According to the Fig. 3b in Ref. 38 in the paper, the migration barriers of interstitials in metals for carbon is in the order of Au < Ag < Cu ≈ Pt < Ru ≈ Co ≈ Pd < Ir < Rh ≈ Ni. It means the carbon diffusivity in the metals is in the following order: Au > Ag > Cu ≈ Pt > Ru ≈ Co ≈ Pd > Ir > Rh ≈ Ni.

Conclusion: Ir & Rh should be excluded due to the slow diffusion.

(3) Form homogenous single-phase solid solution alloy

Ag, Au, Cu, Ir, Ni, Pd, Pt and Rh are FCC metals (e.g. the Cu-Pd system [2] and Au-Ni system [3]).

But Ag-Ni [4], or Ag-Cu [3] are completely immiscible.

Co and Ru are HCP metals. They can dissolve in each other and form complete HCP solid solution [3]. At high temperature, pure Co is stable as FCC, and forms complete solid solution with Ni [3]. However, Co & Cu are immiscible [5].

Finally, Cr is BCC metal and easy to form Cr-carbides, thus Cr should be excluded.

In summary, based on the above concern, we may design the following alloy systems for the low-temperature high-quality growth of graphene.

A) Choose an efficient catalytic metal and forming FCC solid solution with Cu, e.g. the Cu-Pd binary.
B) Choose a fast diffuse metal for carbon and forming FCC solid solution with Ni, e.g. the Au-Ni binary.
C) Try the HCP Co-Ru binary with decent catalytic, diffuse and solubility values.

References

1. Blake, P.; Hill, E. W.; Neto, A. H. C.; Novoselov, K. S.; Jiang, D.; Yang, R.; et al. Making graphene visible. *Appl. Phys. Lett.* **2007**, 91, 063124.
2. Li, M.; Du, Z.; Guo, C.; Li, C. A thermodynamic modeling of the Cu–Pd system. *CALPHAD* **2008**, 32, 439-446.
3. Singleton, M. F.; Murray, J. L.; Nash, P.; in T.B. Massalski (ed.). *Binary Alloy Phase Diagrams*, 2nd ed.; ASM International: Materials Park, OH, USA, 1990; ISBN: 978-0-87170-403-0.
4. Singleton, M.; Nash, P. The Ag-Ni (Silver-Nickel) System. Journal of Phase Equilibria and Diffusion **1987**, 8, 119-121.
5. Nishizawa, T.; Ishida, K. The Co-Cu (Cobalt-Copper) System. Bulletin of Alloy Phase Diagrams 1984, 5, 161–165.
